# Supplementary material for: The Biobanque québécoise de la COVID-19 (BQC19)—A cohort to prospectively study the clinical and biological determinants of COVID-19 clinical trajectories
Source: PLoS One. 2021 May 19;16(5):e0245031. doi: 10.1371/journal.pone.0245031 (PMC8133500; doi:10.1371/journal.pone.0245031)
Supplement: S1 File — (DOCX) [file pone.0245031.s001.docx]

**S1 File**

**Title**

The Biobanque québécoise de la COVID-19 (BQC19) – A cohort to prospectively study the clinical and biological determinants of COVID-19 clinical trajectories

**Authors’ names**

Karine Tremblay, Ph.D., Simon Rousseau, Ph.D., Ma’n H. Zawati, Ph.D., Daniel Auld, Ph.D., Michaël Chassé, MD, Ph.D., Daniel Coderre, Ph.D., Emilia Liana Falcone, MD, Ph.D., Nicolas Gauthier, Ph.D., Nathalie Grandvaux, Ph.D., François Gros-Louis, Ph.D., Carole Jabet, Ph.D., Yann Joly, DCL, Daniel E. Kaufmann, MD, Catherine Laprise, Ph.D., Catherine Larochelle, MD, Ph.D., François Maltais, MD, Anne-Marie Mes-Masson, Ph.D., Alexandre Montpetit, Ph.D., Alain Piché, MD, M.Sc., J Brent Richards, MD, Sze Man Tse, MDCM, MPH, Alexis F. Turgeon, MD, M.Sc., Gustavo Turecki, MD, Ph.D., Donald C. Vinh, MD, Han Ting Wang, MD, MSc, Vincent Mooser, MD on behalf of BQC19.

**Appendix -1- Consent**

More specifically, if a **potential participant is capable,** according to the clinical team, of consent to participate in the BQC19, the consent process may begin. The consent procedure follows these steps: a) the research staff presents BQC19 verbally (by telephone or other electronic tool available in the participant's room) and answers all of the participant's questions; b) the research staff records and/or documents the participant's verbal consent (electronic conversation or other) in the informed consent form (ICF) (electronic version); c) the research staff forwards a copy of the ICF attesting the participant's consent to BQC19, preferably by e-mail or by mail if e-mail is not possible; d) the participant responds to this mailing (electronically or by mail) by including the signature section of the ICF in order to document his or her consent; e) if the participant dies before a response is received or if research staff does not receive a return reminder, the recorded and documented verbal consent is valid.

If a **potential participant is incapable** to provide consent to participate in BQC19 or suddenly becomes incapable for any reason: a) the research staff responsible for BQC19 in the institution contacts the legal representative (mandatary, curator or tutor for the adult who is incapable and spouse, close relative or interested person) by telephone and presents BQC19; b) if the legal representative shows an interest in the participation of the person represented, a copy of the BQC19’s ICF is sent to him/her by e-mail or mail; c) a telephone conversation or videoconference is held to answer questions from the legal representative, if any of the following occurred: a verbal consent is recorded and documented in the ICF (electronic version); d) a copy of the ICF attesting consent is sent to the legal representative by e-mail or mail; e) the legal representative responds to this email or mail by including the signature section of the ICF in order to document his or her consent. A similar process is followed for the recruitment of minors, except the representative in that case will be the person that holds parental or guardianship authority. It is worthy to note that in Quebec law, minors that are 14 years and older could consent alone to research projects that involve minimal risk. For the purposes of BQC19, the competent REB deemed the research to involve more than a minimal risk, hence requiring any minor with less than 18 years of age to have a representative consent. Minors and adult participants incapable of giving consent may not participate in such research where they understand the nature and consequences of the research and object to participating in it.

Moreover, in the case of verbal consent prior to temporary incapacity, if the participant dies prior to a response from the legal representative, or if there is no return call to the research staff, the recorded and documented verbal consent is valid. Finally, if the participant has regained capacity and is required to confirm participation in the BQC19, the procedures for obtaining consent from the participant who is able to consent to the BQC19 described above are followed.

**Appendix -2- BQC19 Blood Samples Processing Standard Operating Procedure**

Depending on the possibilities for the blood samples to be processed, we collect 1 PAXgene® RNA tube, 4 ACD tubes and 1 red-capped tube (serum): to perform DNA profiling, RNA profiling, to isolate plasma and Peripheral blood mononuclear cells (PBMCs) and to collect serum.

All tubes kept at RT before processing; the faster after blood draw the better, ideally <6 hours; <12 hours fine for most assays; >12h: a number of functional assays will become less reliable. Please indicate in the comment section on retrieval the time between treatment and venipuncture.

**Solution preparation**

HEPES 1M

1. 5ml aliquots and store at 4^0^C.

Penicillin Streptomycin (10 000U/ml)

1. 5ml aliquots ans store 4^0^C or -20^0^C.

FBS decomplemented

1. Heat at 56^0^C for 30 minutes to decompliment.

2. 50 ml aliquot and store -20^0^C.

3. Thaw before use.

R+

1. Take a 50ml bottle of RPMI 1640.

2. Add 5mL of HEPES 1M.

3. Add 5mL of Pen/Strep 10000U.

4. Can be stored at 4^0^C.

R10FBS

1. Take 45ml of prepared R+ medium.

2. Add 5mL of decomplemented FBS.

FBS 20%DMSO :

1. Take 40ml of decomplemented FBS.

2. Add 10mL of DMSO.

3. Store at 4°C for 1 week.

4. It will be used as a freezing medium.

5. Note the date on which the solution is prepared.

**Procedure**

### PAXgene RNA

1. Allow the tube to stand at room temperature overnight. Record the date and time on the worksheet (BQC19 – Biobanking sample logsheet).

2. After overnight at room temperature, transfer PAXgene® RNA tubes at -20°C for 24 hours. Record the date and time on the worksheet (BQC19 – Biobanking sample logsheet).

3. Transfer to -80°C for long term storage. Record date, time and location on the worksheet (BQC19 – Biobanking sample logsheet).

### SERUM: serum red tube

1. Centrifuge serum red cap tube at 2000g for 10min at RT BRAKE ON.

2. Recuperate serum in 15mL tube. BE CAREFUL not to disturb the base / clot in the bottom of the tube.

3. Aliquot the serum in volumes of 250µl and volumes of 500µl in the screwcap tubes. For work optimization, this step can be done during the whole blood centrifugation.

4. Complete the worksheet (BQC19 – Biobanking sample logsheet).

### Whole BLOOD: ACD tubes

1. Transfer blood from ACD tubes into 50mL Falcon tube (pooled) usual volume is approximately 30 mL. Record the volume collected on the worksheet (BQC19 – Biobanking sample logsheet).

2. Transfer aliquot of 500µL of whole blood into cryotubes.

### Plasma: Tubes ACD

3. Centrifuge whole blood tubes at 850 g for 10 min, at RT, BRAKE OFF.

4. Remove the plasma layer with plastic transfer pipet (10 mL) and transfer into a 15mL tube.

5. Transfer the plasma into tubes with screw caps, containing 500µL and 250µL/tube.

6. Store tubes at -80°C and note their location in the worksheet (BQC19 – Biobanking sample logsheet).

###

### Ficoll-Hypaque Overlay Method for PBMC isolation

*1.* After Plasma collection, top up blood to 30 mL with HBSS+ medium (30mL total). We need to dilute blood in 1:1 ratio. Mix gently and thoroughly.

2. Take 50 mL tubes with 15mL Ficoll. Plan 2 parts diluted blood for one part of Ficoll (usually 15 ml of Ficoll and 30mL of blood).

3. Carefully and slowly pipette blood on top of Ficoll solution in 50 mL centrifuge tubes (gently allow mixture to flow down along the side of tube).

4. Centrifuge tubes at RT for 30 minutes at 400g BRAKE OFF. Handle carefully and make sure that tubes are balanced to not disrupt layering

5. After Ficoll separation, gently collect the PBMC layer by aspirating it using a plastic Pasteur pipette. Start from about 1 mm away, beginning by the sides of the tube. Transfer the cells to a clean 50ml tube (maximum 20ml/tube).

6. First wash: Top up cells suspension to 45mL with HBSS. Centrifuge at 400g, for 10 min, BRAKE ON.

7. Decant media after centrifuging.

8. Second wash: Use a 1ml in pipette tips to break up pellet. Wash again adding 44mL R+. Spin at 400g, for 10 min, BRAKE ON.

9. Decant media and resuspend cells in about 5 mL of R10FBS. Set centrifuge at 4°C.

###

### ALTERNATIVE: SepMate Method for PBMC isolation

1. After Plasma collection, top up blood to 30 mL with PBS-2%FBS (30mL total). Mix gently and thoroughly.

2. Take 2 SepMate 50 mL tubes per sample and fill it carefully by pipetting it through the central hole with 15mL Ficoll.

3. Keeping the SepMate vertical, add the 15mL diluted blood by pipetting it down the side of the tube (2 tubes per sample).

4. Centrifuge tubes at RT for 10 minutes at 1200g brake OFF. Handle carefully and make sure that tubes are balanced to not disrupt layering.

5. After centrifugation pour off the top layer of into a new tube by inverting tube not more longer than 2 seconds.

6. First wash: Top up cells suspension to 45mL with PBS-2%FBS Centrifuge at 300 g for 8 min, brake ON.

7. Second wash: Top up cells suspension to 45mL with PBS-2%FBS Centrifuge at 300 g for 8 min, brake ON.

8. Decant media and resuspend cells in about 5 mL of R10FBS. Set centrifuge at 4°C.

### PBMC COUNT

1. *Manual cell counting* Count and record the number of viable PBMCs per mL. If done via an automatic cell counter, follow the supplier's instructions.

### Freezing PBMCs

1. Count cells to be frozen and keep in fridge until ready to spin and freeze. Write number on SOP blood processing log sheet (BQC19 – Biobanking sample logsheet).

2. Note: we usually freeze down 10M PBMC per vial minimum for storage in nitrogen tank. Low number of cells per tube leads to lower relative recovery in terms of cell number.

3. Spin cells (from previous step 8) in a cold centrifuge (4°C) for 10 minutes at 400g.

4. After the spin, aspirate supernatant until 100uL of residual volume is left and resuspend pellet.

5. Resuspend PBMC in cold pure decomplemented FBS at 20M/mL.

6. Add freezing solution (20% DMSO FBS) 1:1 (same volume as FBS at step 5) drop-by-drop while CONTINUOUSLY shaking the tube.

7. Transfer 1 mL to each labeled Nalgene cryovial.

8. Once cells are in freezing solution, place cells in Mr Frosty box inside the -80°C freezer.

9. Do not keep the vials containing cells and freezing solution on ice for too long before they are placed in the -80°C freezer. DMSO is toxic to cells, so their viability will suffer if they are not frozen quickly enough. Do not prepare too many tubes simultaneously if you lack experience.

10. Cells transferred to LN_2_ the next day.

**Appendix -3- Access**

Applicants must be Canadian and international researchers from academia, hospitals or private entities. Access is granted through a fair, non-discriminatory, objective and transparent process. To that end, clear rules for access are published on the BQC19 website. Data access is subject to permissions, legal and regulatory frameworks, and is dependent on the nature of the requested data. Obtaining data or biosamples is also subjected to applicable cost recovery access fees. Requests are evaluated according to the following criteria: i) the value of the data to be returned to BQC19; ii) the scientific contribution of the research project; iii) the potential impact of sample access on the risk of depletion of the biobank; iv) adherence to the FAIR (Findable, Accessible, Interoperable, Reusable) principles (6). The use of the biobank resources should serve to maximize scientific, clinical and societal benefits. The use of limited and precious biosamples should be supported by demonstrated scientific validity that accords with the BQC19 mission and must be supported by sufficient technical expertise to carry out the proposed analysis. The use of limited and precious biosamples should be supported by demonstrated scientific validity that is aligned with the BQC19 mission and must be supported by sufficient technical expertise and funding to carry out the proposed analysis. Thus, the access process should demonstrate and continuously improve the value, utility and sustainability of BQC19. It should also maintain the reputation of BQC19 and its participating institutions and funders. Any experimental data made possible by access must be shared with BQC19 and made available to third parties, in accordance with the Statement on data sharing in public health emergencies (https://wellcome.org/coronavirus-covid-19/open-data). Each request for access to BQC19 resources is assessed by an access committee formed of reviewers that are independent from BQC19 and free of conflicts-of-interest. The controlled access process is initiated by the submission of a request via the access portal on the BQC19 website. The steps in the application process are presented in **Figure 5**.

Finally, given the mission of BQC19, researchers from academia, the Quebec public health network or the private sector, in Canada and internationally, will be permitted to access BQC19 biological materials and data whose research projects meet appropriate scientific and ethical standards.
